# How do lipids influence risk of violence, self-harm and suicidality in people with psychosis? A systematic review

**DOI:** 10.1177/00048674211025608

**Published:** 2021-07-09

**Authors:** Piyal Sen, Danielle Adewusi, Alexandra I Blakemore, Veena Kumari

**Affiliations:** 1 Department of Life Sciences, Centre for Cognitive Neuroscience, College of Health, Medicine and Life Sciences, Brunel University London, Uxbridge, UK; 2 GKT School of Medicine, Faculty of Life Sciences & Medicine, King’s College London, London, UK; 3 Department of Metabolism, Digestion and Reproduction, Imperial College London, London, UK

**Keywords:** Cholesterol, aggression, suicide, schizophrenia, sex

## Abstract

**Objectives:**

Low cholesterol has been linked with violent and suicidal behaviour in people with schizophrenia. This association, if consistently present, may be a promising biological marker that could assist clinicians in decision making regarding risk and treatment. We conducted a systematic review to assess whether there is a reliable association between lipid profile (total cholesterol, high- and low-density lipoprotein cholesterol, and triglycerides) and aggression, self-harm or suicide in people with schizophrenia, and whether effects are similar in males and females.

**Method:**

Relevant databases were searched to identify primary research studies (up to November 2020) that (1) involved adults (some samples also included 16- to 18-year olds) with a confirmed diagnosis of schizophrenia, schizoaffective disorder or psychosis; and (2) included a standardised assessment of verbal aggression, physical aggression against objects, physical aggression against self (including suicide) or others. The search yielded 23 studies eligible for inclusion following a quality appraisal.

**Results:**

Suicidality was the most commonly assessed subtype of aggression (20 studies). For suicidality, about half the studies, including the study with the largest sample size, found a link with total cholesterol. An association between low total cholesterol and violence towards others was found in six of nine studies that investigated this. The evidence for a link with violence was the strongest for total cholesterol, followed by low-density lipoprotein cholesterol and high-density lipoprotein cholesterol, and the weakest for triglycerides. Only a few studies investigated sex differences and yielded mixed evidence. Studies focussed on self-harm as well as involving females in forensic settings were lacking.

**Conclusion:**

There is encouraging evidence of an association between low total cholesterol and aggression towards others as well as suicidality in schizophrenia. Future studies should systematically explore this association in people with schizophrenia who have a significant history of violence, suicidality and self-harm, both inpatients and community, and also investigate underlying mechanisms.

## Introduction

The majority of patients in forensic psychiatric services have schizophrenia (Fleischman et al., 2014; Silverstein et al., 2015) or personality disorder (PD), often presenting co-morbidly (Arseneault et al., 2000). There is considerable evidence of an association between interpersonal violence and schizophrenia (Fleischman et al., 2014; Silverstein et al., 2015; Walsh et al., 2002). Violent crime has been shown to be up to 10 times more common in people with schizophrenia than matched populations (Fazel et al., 2009). A growing body of evidence (review, Sedgwick et al., 2016) suggests that several neurobiological measures are aberrant in forensic psychiatric populations. Some of these aberrations, if reliably associated with risk or outcomes, could assist clinicians to make decisions about treatment planning, risk and discharge (Sedgwick et al., 2016). One such promising biological marker could be the lipid profile, particularly serum cholesterol.

A link between low levels of cholesterol and violent behaviour in forensic psychiatric populations has been suggested for over 25 years (Boston et al., 1996; Douglas and Nasrallah, 2019; Sedgwick et al., 2016). Low cholesterol may also be linked to suicidality (Asellus et al., 2010; Lee and Kim, 2003; Wu et al., 2016). Low cholesterol has been discussed as a biomarker for suicide since the early 1990s when meta-analytic evidence associated the use of statins (cholesterol-lowering drugs) with an elevated risk of death by suicide, despite these drugs lowering the risk of death by coronary events (Muldoon et al., 1990). A significant relationship between low serum cholesterol levels and suicide in psychiatric disorders, including psychosis, has also been reported (Kułak-Bejda et al., 2021). Furthermore, low cholesterol has been linked to inpatient suicidal and violent behaviour, and with 3-month post-discharge violent behaviour in non-forensic patient populations (Roaldset et al., 2011), as well as with criminal violence in the general population (Golomb et al., 2000). However, some studies indicate that low cholesterol-aggression association may be true only for men (Tomson-Johanson and Harro, 2018), and a few studies failed to observe this association (Deisenhammer et al., 2004; Roy et al., 2001). To what extent this association is influenced by sex or reliably present in people with schizophrenia remains unclear.

The main aim of this review, therefore, was to systematically investigate possible associations between lipid profile, as indexed by the levels of total cholesterol (TC), high-density lipoprotein cholesterol (HDL), low-density lipoprotein cholesterol (LDL) and triglycerides (TG), and specific dimensions of aggression and violence in adult patients with schizophrenia. For the purpose of this review, aggression was defined as any behaviour falling under verbal aggression, physical aggression against objects, physical aggression against self (including suicide) or physical aggression against others, in line with the Modified Overt Modified Overt Aggression Scale (MOAS) (Sorgi et al., 1991). Our secondary aim was to consider potential sex differences in these associations wherever possible.

## Methods

### Information sources and search

We followed the guidelines detailed in the Preferred Reporting Items for Systematic Reviews and Meta-Analysis (PRISMA) (Moher et al., 2009) for literature search. An extensive search of PubMed, PsycINFO, Academic Search Complete, CINAHL Plus, MEDLINE, Scopus and Web of Science databases was carried out in the first week of December 2020. A combination of the following search terms was used: (cholester* OR high density lipoprotein OR HDL OR low density lipoprotein OR LDL OR triglycerides OR lipid profile) AND (schizoph* OR psychosis OR psychotic OR schizoaffective*) AND (violen* OR aggress* OR assault OR suicide* OR self-harm* OR self-mutilation). Other studies were located through hand searching of relevant publications and reference lists.

### Screening and selection

Articles were selected using the following pre-formed inclusion and exclusion criteria: (1) patients must have a diagnosis of schizophrenia, schizoaffective disorder or psychosis stated through either a DSM (*Diagnostic and Statistical Manual of Mental Disorders*) or ICD (International Classification of Diseases) classification; (2) participants must be adults, aged 18 years and over, unless unavoidable (i.e. studies where the vast majority of participants were over 18, but included some participants between 16 and 18 years, were included); (3) aggressive behaviour described must fall under at least one type stated in the MOAS – verbal, physical against objects, physical against self, physical against others; (4) level of cholesterol must be specified with units; (5) access to full published text and methodology must be available (excluding review articles, editorials and data from conference publications); and (6) articles must be available in English. Two blinded reviewers (P.S. and D.A.) independently made the study selection according to these criteria. Both reviewers had to agree with the articles selected.

### Quality appraisal and data extraction

The quality of the selected studies was then assessed using The Joanna Briggs Institute (JBI) Critical Appraisal Tools for cross-sectional, case–control and case series studies (Zeng et al., 2015). One point was granted for meeting each of the criteria. Articles scoring less than 80% were excluded. Each criterion was graded as ‘yes’, ‘no’, ‘unclear’ or ‘not applicable’. One point was granted for scoring ‘yes’ on each of the criteria, and then all points added to derive the total score, expressed in percentage, for each study. Studies assessed ranged in score from 71.4% to 100% (Tables 1–3). Articles scoring less than 80% were excluded. The mean score of papers included in the review following quality appraisal was 94.2%.

**Table 1.** The Joanna Briggs Institute (JBI) quality appraisal ratings for included and excluded studies: case–control studies.

Study	Study design	Comparable groups other than the presence or absence of the disease	Appropriate matching of cases and controls	Same criteria used for identification of cases and controls	Standard, valid and reliable measurement of exposure	Exposure measured in same way for cases and controls	Identification of confounding factors	Strategies to deal with confounding factors stated	Outcomes assessed in a standard, valid, reliable way	Exposure period of interest long enough to be meaningful	Appropriate statistical analysis used	Score (%)	Overall appraisal
Turkoglu et al. (2009)	Case control. Categorical observation between schizophrenia patients with and without a record of crime	Participants all male Comparable in age: mean (SD) of crime group = 36.52 (8.48), mean (SD) of control group = 35.36 (7.74)	50 male schizophrenia patients with at least one crime record matched with 50 male schizophrenia patients without a crime record. No significant differences in mean age, height or weight between groups	Schizophrenia diagnosis made according to DSM-IV in both cases and controls	Blood samples for TC, TG and ghrelin collected between 08.00 a.m. and 11.00 a.m. TC and TG levels were measured by Olympus Biochemical Autoanalyser, by spectrophotometric method using Olympus brand kits	Blood samples collected and examined for all subjects in the case and control group	Medication and diet identified as potential confounders	No strategy for dealing with confounders	Patients grouped into those with a criminal record and without a criminal record	Blood samples collected between 08.00 a.m. and 11.00 a.m.	*t*-test used	90	Include
Marcinko et al. (2005)	Case control. Categorical observation between schizophrenia patients with violent, non-violent and no suicidal attempts (control group)	Participants all male. No significant differences in age, BMI, marital, family or employment status	31 male schizophrenia patients with a suicide attempt matched with 15 male schizophrenia patients with no history of suicide	Schizophrenia diagnosis made according to ICD-10 in both cases and controls	Blood samples were collected at 7 a.m. after overnight fasting. TC determined enzymatically, immediately after blood collection. Assays done with commercial kits on Olympus AU 600 automatic analyser	Blood samples were collected from all subjects in the case and control group	Age and BMI identified as confounders	Confounders controlled in ANCOVA analysis	Patients grouped into those admitted following a suicide attempt and those without a history of suicide attempt	Blood samples collected after overnight fasting	ANOVA and ANCOVA used	100	Include
Chakrabarti et al. (2004)	Case control. Categorical observation between psychotic patients with a history of violent crime and without a history of violent crime	Participants all male diagnosed with a psychotic illness	30 inpatients with a psychotic illness with a history of violent crime matched through age, sex and BMI to 30 male psychotic inpatients without a history of violent crime	All psychotic illnesses diagnosed according to ICD-10 in both cases and controls	Blood sample obtained after a 12-hour overnight fasting. Biochemical assay used. TC, TG triglycerides and HDL-cholesterol were estimated by colorimetric method. VLDL and LDL cholesterol were calculated from Friedewald and Fredrickson’s formula	Blood sample obtained from each person selected for the study	Medication identified as potential confounder	No strategy for dealing with confounders	Patients grouped into those with a history of violent crime, which included homicide, rape, arson and grievous injury as per the Criminal Procedure Code of the Indian Law, and those without a history of violent crime	Blood sample obtained after a 12-hour overnight fasting	Pearson correlation coefficients used	90	Include
Tripodianakis et al. (2002)	Case control. Categorical observation between patients and healthy controls (no psychiatric history), matched for age and sex	Suicide and non-suicide attempt groups were age matched according to sex – male controls were age matched to male attempters and female controls matched to female attempters	Case group, admitted after suicide attempt, consisted of 35 males and 76 females. Control group, with no psychiatric history or history of suicide attempt, consisted of 31 males and 31 females. Ages of control subjects matched according to sex	n/a – control group did not have a psychiatric diagnosis	Blood and urine samples taken within 24 hours of admission at 8:00 in the morning. Total cholesterol was estimated in plasma samples using commercially available kits	Blood and urine samples taken within 24 hours of admission from all the patients	Age identified as confounder	Confounders controlled in ANOVA	Severity of the suicidal intent was estimated with SIS	Blood and urine samples were taken within 24 hours of admission	ANOVA and linear regression analysis used	100	Include
Repo-Tiihonen et al. (2002)	Case control. Categorical observation between patients who had been secluded involuntarily (for violent acts, for suicidal acts) and patients who had not been secluded	Participants all males diagnosed with a psychotic illness	None stated	All psychotic illnesses diagnosed according to ICD-10 in both cases and controls	The serum samples for assaying TC levels were collected always in the morning after 10 to 12 hours’ fast. The method for assaying was enzymatic	Blood samples taken in the morning after 10–12 hour fast	States that BMI did not explain differences in TC, suggesting that this may have been a potential confounder	No strategy for dealing with confounders	‘Violent and dangerous’ psychiatric patients sent to his hospital by municipal psychiatric hospitals	Blood samples taken in the morning after 10–12 hour fast	Mann–Whitney *U*-test used	80	Include
Mufti et al. (1998)	Case control	All participants admitted during the same 2-month period in early 1995	Case group consisted of 17 African Americans and control group consisted of 16 African Americans	Not specified	Method of measuring TC and TG not stated	TC and TG obtained from medical chart review for all patients	Age, sex and race identified as confounders	Confounders controlled in ANCOVA and logistic regression	Patients grouped into those who were violent, were secluded or restrained, and those who did not receive seclusion or restraint	Seclusion or restraint within 2-month period	ANCOVA and logistic regression used	80	Exclude on the basis of lack of recognised diagnostic classification system

SD: standard deviation; DSM-IV: *Diagnostic and Statistical Manual of Mental Disorders* (4th ed.); TC: total cholesterol; TG: triglyceride; BMI: body mass index; ICD-10: International Classification of Diseases, 10th Revision; ANCOVA: analysis of covariance; ANOVA: analysis of variance; HDL: high-density lipoprotein; VLDL: very low-density lipoprotein; LDL: low-density lipoprotein; SIS: Scale for Impact of Suicidality.

**Table 2.** The Joanna Briggs Institute (JBI) quality appraisal ratings for included and excluded studies: Case series studies.

Study	Study design	Clear criteria for inclusion in case series	Condition measured in standard, reliable way for participants in case series	Valid methods used for identification of condition for participants in case series	Consecutive inclusion of participants	Complete inclusion of participants in case series	Clear reporting of demographics of participants	Clear reporting of clinical information of participants	Outcomes or follow-up results of cases clearly reported	Clear reporting of presentation site/clinic demographic information	Appropriate statistical analysis	Score (%)	Overall appraisal
Chen et al. (2015)	Case series. Correlational observation.	Inclusion criteria: schizophrenia patients over the age of 16 who were admitted to the acute psychiatric ward in National Taiwan University Hospital	DSM-IV criteria used Psychotic symptoms assessed weekly by psychiatrists using the Comprehensive Psychopathological Rating Scale for inpatients with severe mental disorder	DSM-IV classification used.	Patients were recruited in succession from April 2002 to March 2003	Yes, *n* = 107	Age, gender, education, unemployment and being single all reported	Positive symptoms scores, negative symptoms scores, age at onset and BMI reported	Tables reporting relationships between schizophrenia and violence and explained in text. *p* values reported	National Taiwan University Hospital. Demographic information not specified	Logistic regression analysis used	90	Include
Ruljancic et al. (2007)	Case series. Categorical observation between psychiatric patients	Inclusion criteria: all patients admitted to Sveti Ivan Psychiatric Hospital were included. Exclusion criteria: Patients with psychiatric diagnosis of low prevalence were excluded	ICD-10 criteria used	ICD-10 classification used	All patients admitted from January 2005 to April 2005	Yes, *n* = 677	Age: 17–89 262 females, 415 males Geographic region: Zagreb, Croatia	Schizophrenia, *n* = 136 Schizoaffective disorder, *n* = 64 Psychotic disorders, *n* = 47	Table shows mean and SD of TC in patients and explained in text. *p* values reported	Sveti Ivan Psychiatric Hospital, Zagreb, Croatia. Demographic information not specified	ANOVA test used	90	Include
Huang et al. (2000)	Case series. Categorical analysis – between paranoid and non-paranoid schizophrenic patients	Inclusion criteria: all patients admitted to the acute psychiatric inpatient unit in the Chang Gung Memorial Hospital Exclusion criteria: no comorbid illness	DSM-III-R criteria used	DSM-III-R classification used	All patients admitted from January to December 1995	Yes, *n* = 213	Mean age = 10.8 47 males, 59 females Geographic region: Kaohsiung, Taiwan	Mean BMI = 23 Paranoid and non-paranoid schizophrenia, *n* = 106	Table shows mean and SD of TC in patients and explained in text. *p* values reported	Chang Gung Memorial Hospital in Kaohsiung, Taiwan. Demographic information not specified	*t*-test used	90	Include

DSM-IV: *Diagnostic and Statistical Manual of Mental Disorders* (4th ed.); BMI: body mass index; ICD-10: International Classification of Diseases, 10th Revision; SD: standard deviation; TC: total cholesterol; ANOVA: analysis of variance; DSM-II-R: *Diagnostic and Statistical Manual of Mental Disorders* (2nd ed., rev.).

**Table 3.** The Joanna Briggs Institute (JBI) quality appraisal ratings for included and excluded studies: cross-sectional studies.

Study	Study design	Inclusion criteria clearly defined	Study subjects and setting described in detail	Exposure measured in a valid and reliable way	Objective, standard criteria used for measurement of condition	Confounders identified	Strategies to deal with confounders	Outcomes measured in a valid way	Appropriate statistical analysis	Score (%)	Overall appraisal
Sankaranarayanan et al. (2020)	Cross-sectional	Not clearly defined. Descriptive characteristics and ICD classification given but no distinct inclusion criteria	Age, sex, education, employment and marital status given. Sample collected from a database of Australians with psychotic disorders	Fasting levels of TC, HDL, LDL and TG were collected and analysed	ICD-10 classification of schizophrenia and schizoaffective disorder used	Age, sex, smoking status, cannabis use, dysphoria and BMI identified as potential confounders	Potential confounders controlled in logistic regression	Suicidal ideation assessed using the Diagnostic Interview for Psychosis	Multivariable logistic regression used	87.5	Include
Hjell et al. (2020)	Cross-sectional	Inclusion criteria: a diagnosis within the schizophrenia or bipolar spectrum, age between 18 and 65 years, ability to give informed consent, and Norwegian language skills sufficient for valid assessments. Exclusion criteria: marked cognitive deficit (IQ scores below 70), neurological disorder and history of severe head trauma	Age, sex, race, smoking status and medication given. Study formed part of the TOP study, where patients with severe mental disorders were recruited from psychiatric inpatient and outpatient clinics of the major hospitals in Oslo, Norway	Venous blood samples were collected in the morning after an overnight fast of at least 8 hours. Levels of TC, LDL and HDL were measured	DSM-IV classification of psychiatric disorders used. The Positive and Negative Syndrome Scale (PANSS) used to measure severity of symptoms	Age, sex, BMI and medication identified as confounders	Confounders controlled in logistic regression	Aggression measured using PANSS Excited Component. Impulsivity measured using Barratt Impulsiveness Scale	Multinomial logistic regression used	100	Include
Gohar et al. (2019)	Cross-sectional (with correlational analysis)	Inclusion criteria clearly defined: 18–65 years of age, DSM-IV criteria for schizophrenia and psychotic disorders, have available serum leptin measurements and able to provide consent to participate. Participants with a history of significant head injury, neurological disorder, mental retardation and autoimmune disease were excluded	Age, sex, ethnicity, education, smoking status and age of onset recorded. Subjects were recruited through the ongoing Norwegian TOP study	Fasting venous blood samples were obtained in the morning. Measurement and analysis of TC, TG, LDL and standard C-reactive protein were conducted	DSM-IV classification of psychiatric disorders used. The Structured Clinical Interview for DSM-IV Axis I Disorders (SCID-I) used to confirm the diagnosis	Age, sex, BMI, dietary habits, smoking, age of onset and duration of illness identified as confounders	Confounders controlled in logistic regression	Severity of suicidal behaviour measured using item 18 of IDS-C	Multinomial logistic regression used	100	Include
Goel et al. (2019)	Cross-sectional	Inclusion criteria clearly defined: patients of either sex and those patients or accompanying relatives willing to give written informed consent for participation in study. Exclusion criteria: patients having comorbid physical disorders	Age, sex and marital status recorded. Patients recruited from inpatient and outpatient of AVBR Hospital	Blood sample for total cholesterol level was taken immediately after admission in psychiatry ward or on OPD basis	No DSM or ICD classification used for diagnosis	Mental illness, medication and dietary factors were identified as confounders	Confounders not handled within study	Patients grouped into recent suicide attempt, suicidal ideations but no attempts and no suicidal ideation or attempt	Chi-square test used	75	Exclude
Shafti et al. (2019)	Cross-sectional	Inclusion criteria clearly defined: all registered inpatients with suicidal behaviour (successful suicide and attempted suicide, in total), during the last 60 months	Age, sex, marital status and employment recorded. Setting described in detail: Razi psychiatric hospital, as the largest psychiatric hospital in the Middle East, five acute academic wards, which are specified for admission of first episode adult psychiatric patients, and five acute non-academic wards, specified for admission of recurrent episode adult psychiatric patients	Serum lipids, including TC, TG, LDL and HDL were analysed, having being collected as part of routine laboratory tests for all patients upon admission	DSM-IV classification of psychiatric disorders used	Confounders, such as age, sex, BMI and involuntary admission, were not identified	No strategy to deal with confounders	Retrospective evaluation identified in patients with suicidal behaviour (successful suicide and attempted suicide, in total)	*T*-test used	75	Exclude
Cariou et al. (2018)	Cross-sectional	Inclusion criteria clearly defined: admitted in the Psychiatry department in the course of the year 2014, to be aged 18 and older, and to have had a lipid panel test Exclusion criteria: patients admitted in the eating disorders unit	Age, sex, weight, height, BMI, medication, history of diabetes, liver diseases and cardiovascular risk factors recorded. Recruited from the psychiatry department of Nantes University Hospital, France	Lipid panel test was done and TC, HDL, and TG were collected and analysed. LDL was calculated according to the Friedewald formula.	ICD-10 classification of psychiatric disorders used	Medical cause of HBL identified as confounder	Patients with medical cause of HBL excluded	Hetero-aggression, suicidal attempts and other self-injuries categorised as aggressive behaviours	Mann–Whitney tests for the quantitative variables and Fisher tests for the qualitative variables	100	Include
Eriksen et al. (2018)	Cross-sectional with longitudinal component	Inclusion criteria: all patients admitted to the acute psychiatric ward at Oslo University Hospital, Norway, between 21 March 2012 and 20 March 2013	Age, gender, employment, education, marital status recorded. Acute psychiatric ward at Oslo University Hospital admits adults (18 years or older) from a catchment area of 204,000 people	Serum measures of TC and HDL in millimoles per litre (mmol/L) were obtained from routine blood tests at admission and analysed with ‘enzymatic colorimetric method’ for TC and ‘homogeneous enzymatic colorimetric method’ for HDL (‘Cobas 8000 c702’, Roche Diagnostics, Oslo, Norway)	ICD-10 classification of psychiatric disorders used	Involuntary admission and age identified as confounders	Confounders controlled in logistic regression	Violent behaviour measured using SOAS-R. Association between cholesterol and suicide attempt measured using appropriate statistical analysis	Uni-, bi- and multivariate binary logistic regression analyses used	100	Include
Capuzzi et al. (2018)	Cross-sectional	Inclusion criteria: patients admitted between January 2012 and December 2017, to Psychiatric Inpatient Unit (Desio Hospital, ASST Monza, Italy), psychiatric diagnosis classified by ICD-10, aged between 18 and 65 years, medically stable, not needing treatment for any physical condition. Exclusion criteria: subjects suffering from other mental disorders or mental retardation, serious physical illnesses or treated with thyroid hormone, antidiabetic, anticoagulant, anti-platelet, urate- and lipid-lowering agents	Age, sex, smoking status, BMI, and medication recorded Psychiatric Inpatient Unit at Desio Hospital, ASST Monza, Italy	Information on TC, LDL and TG retrieved from routine blood samples drawn at approximately 8.00 a.m., after an overnight fasting. Blood tests carried out within 24 hours after hospitalisation	ICD-10 classification of psychiatric disorders used DSM-IV classification of schizophrenia used	Age and sex identified as confounders	Confounders controlled in logistic regression	Standard definitions used to distinguish violent (firearm, hanging, cutting, jumping, car exhaust, other violent methods) from non-violent (drug overdose and poisoning) attempt methods	Logistic regression analyses used	100	Include
Eriksen et al. (2017)	Cross-sectional with a longitudinal component	Inclusion criteria: all patients admitted from 21 March 2012 to 20 March 2013 from Oslo University Hospital Exclusion criteria: incomplete serum-lipid samples	Age, sex, involuntary admission, employment, education status and marital status recorded Acute psychiatric ward at Oslo University Hospital has five units (45 beds) for emergency psychiatric admissions from a catchment area of about 200,000 persons older than 18 years	Serum measures of TC and HDL in millimoles per litre (mmol/L) were obtained from routine blood tests at admission and analysed with ‘enzymatic colorimetric method’ for TC and ‘homogeneous enzymatic colorimetric method’ for HDL	ICD-10 classification of psychiatric disorders used	Male gender, involuntarily admitted and psychosis identified as potential confounders	Confounders controlled in logistic regression	Violent behaviour measured using SOAS-R Association between cholesterol and suicide attempt measured using appropriate statistical analysis	Multivariate binary logistic regression used	100	Include
Shrivastava et al. (2017)	Cross-sectional, cohort study. Categorical observation	Inclusion criteria: age 18–30, schizophrenia diagnosed by DSM-IV-TR, inpatients in the first episode of the illness and consent Exclusion criteria: organic psychiatric conditions, other major psychiatric disorders, substance abuse (excluding nicotine dependence), and major medical or surgical illnesses	Age, sex and duration of illness recorded A tertiary general psychiatric centre (hospital name not provided), in India	TC collected from routine blood investigation. Data TC were retrieved from the biochemistry database of the facility	DSM-IV-TR classification of schizophrenia used	Differences in socioeconomic status, metabolic profiles and dietary differences identified as confounders	No strategy to deal with confounders	SIS-MAP used to measure severity of suicidal behaviour	Pearson correlation analysis used	87.5	Include
Kavoor et al. (2017)	Cross-sectional. Categorical observation compared to age, sex and ethnicity matched healthy controls	Inclusion criteria: schizophrenia diagnosed with ICD-10 classification, antipsychotic-drug naïve or antipsychotic-drug free for at least 4 weeks (oral medication) or 8 weeks (depot medication), educated to at least class 5 Exclusion criteria: diagnosis of substance dependence, other psychiatric or eating disorders, comorbid neurological disorder or medical conditions and taking oral contraceptives and beta blockers	Age, sex, education, religion, marital status, family habitat and BMI recorded Tertiary-level referral centre and a post graduate teaching hospital in western Uttar Pradesh	Venous blood samples (5 mL) were drawn on weekdays between 7 and 9 a.m. after the participants had fasted for at least 12 hours. Samples were immediately delivered to the hospital laboratory and analysed for TC, HDL, LDL, VLDL and TGs using enzymatic auto-analyser	ICD-10 classification of schizophrenia used	Substance abuse, nutritional status and use of historical indices of violence identified as confounders	No strategy to deal with confounders	MOAS, IRS and BSI were used to quantify impulsivity, aggression and suicidality, respectively	Pearson’s correlation analysis used	87.5	Include
Mensi et al. (2016)	Cross-sectional	Unclear inclusion criteria – stated that patients included met the inclusion criteria but did not specify the nature of the criteria	Age, sex, BMI, smoking status and alcoholic status were recorded. Recruited from the University Hospital of Monastir, located in the Mid-eastern part of Tunisia	Venepuncture was performed for all subjects between 8 and 9 a.m. after a 12-hour overnight fast. Approximately 5 mL of blood was collected. Immediately after collecting blood samples, serum concentration of total cholesterol (TC), high-density lipoprotein cholesterol (HDL-c), and triglycerides (TG) were determined using enzyme methods on COBAS 6000 automates analyser, and low-density lipoprotein cholesterol (LDL-c) was calculated with the Friedewald equation	DSM-IV classification of schizophrenia used	Medication identified as a potential confounder	No strategy to deal with confounders	Suicide attempts divided into two groups: lifetime suicide group (who had attempted suicide for more than 2 months) and recent suicide group (who had attempted suicide for less than 2 months)	Chi-square test and independent sample *t* test used	75	Exclude
Misiak et al. (2015)	Cross-sectional cohort study	Inclusion criteria: schizophrenia diagnosis based on DSM-IV and confirmed using OPCRIT checklist, admitted to Lower Silesian Center of Mental Health (Wroclaw, Poland) Exclusion criteria: mental retardation and/or general brain disorder, supplementation of folic acid or vitamins B, medications influencing lipid profile, positive urine screening for illicit drugs, alcohol or drug abuse/dependence during 1 year prior to the onset of psychosis and severe somatic comorbidities	Age, sex. education, marital status, employment, weight, height, and BMI recorded. Lower Silesian Center of Mental Health (Wroclaw, Poland)	Blood samples were obtained between 7.30 and 8.30 a.m. after at least 10 hours overnight fasting from the antecubital vein. TC determined using a Cobas 6000 analyser. Enzymatic methods used also to measure TC, HDL and TG. LDL was calculated using the Friedewald equation	DSM-IV classification of schizophrenia used	Age, BMI, chlorpromazine dosage and treatment duration identified as confounders	Confounders controlled in general linear model	Patients divided into two subgroups based on those who had a lifetime experience of suicidal ideation and those who did not. Subgroups derived based on OPCRIT checklist	General linear model and two-way ANOVA used	100	Include
Ainiyet et al. (2014)	Cross-sectional	Inclusion criteria: schizophrenia diagnosed by at least two psychiatrists according to DSM-IV criteria (SCID), acute exacerbation of schizophrenia Exclusion criteria: severe somatic illness, drug and/or alcohol abuse/dependence, comorbid eating disorder, and using low-fat diet, lipid-lowering drugs or hormonal therapy	Age, sex and duration of illness recorded. Inpatient clinic at the Department of Adult Psychiatry, Poznan University of Medical Sciences	Fasting blood was drawn at 8:00 a.m. within 24–72 hours of admission to the outpatient clinic. Laboratory measures included concentrations of the following lipids: total cholesterol, LDL cholesterol, HDL cholesterol, triglycerides and total lipids	Schizophrenia diagnosed by at least two psychiatrists according to DSM-IV criteria (SCID)	Potential confounder, such as age, smoking, involuntary admission and a previous history of suicide attempt, not identified	No strategy for dealing with confounders	Non-paired *t*-test used		75	Exclude
Park et al. (2013)	Cross-sectional	Inclusion criteria: schizophrenia, bipolar affective disorder or major depressive disorder, based on ICD-10, aged over 18 Exclusion criteria: patients who lacked fasting laboratory values, had a substance use disorder or an eating disorder, or took cholesterol-lowering drugs	Age, sex, number of admissions, BMI, socioeconomic status and comorbid disease recorded. Recruited from a psychiatric ward at a university hospital in Seoul, Korea	TC, HDL and TG were recorded at time of admission	ICD-10 classification of schizophrenia used	Previous history of suicide attempt identified as a potential confounder	No strategy for dealing with confounders	Patients grouped into two categories: patients who died by suicide and patients that did not	Independent *t*-test for continuous variables and a chi-square test for categorical variables	85.7	Include
Marcinko et al. (2008)	Cross-sectional	Inclusion criteria: male schizoaffective patients whose biochemical analyses from the time of admission were available, treated at Department of Psychiatry, University Hospital Zagreb, during the period of 18 months Exclusion criteria: hypertension, hypothyroidism, diabetes mellitus, disorders of the lipoprotein metabolism, diagnosis of substance abuse, including alcoholism, eating disorders and organic brain syndrome	Age, BMI, previous hospitalisation and duration of illness recorded Department of Psychiatry, University Hospital Zagreb	Venepuncture was performed for all subjects between 8 and 9 a.m. after 12-hour overnight fast. Immediately after collecting blood samples, TC, HDL and triglycerides were determined using enzyme method and commercial kits (Olympus Diagnostic, GmbH, Hamburg, Germany) on Olympus AU 600 automated analyser	ICD-10 classification used	Poor physical health, dietary habits, social and economical conditions identified as potential confounders	No strategy for dealing with confounders	Patients suicidal at hospital admission if suicidal ideation, suicide attempt, or both, was present. Suicidality was assessed positive if item 3 of the Hamilton Depression Rating Scale (HDRS-17) scale was scored positively. Severity of suicidality was measured by SSI at the time of admission	Nonparametric Spearman correlation coefficient test used	85.7	Include
Marcinko et al. (2007)	Cross-sectional with longitudinal follow-up over 12 months	Inclusion criteria: male patients diagnosed with Psychotic Disorder Not Otherwise Specified, admitted to Department of Psychiatry, University Hospital Zagreb, during the period of 12 months Exclusion criteria: hypertension, hypothyroidism, diabetes mellitus, disorders of the lipoprotein metabolism, diagnosis of substance abuse, including alcoholism, eating disorders and organic brain syndrome	Age, BMI, family status, Church attending, and family history of suicide or suicide attempt recorded Department of Psychiatry, University Hospital Zagreb	Blood samples were collected from all subjects at 8.00 a.m. after an overnight fasting. TC was determined enzymatically, immediately after the blood collection, using commercial kits	DSM-IV classification used	Age and BMI identified as confounders	Confounders controlled in ANCOVA test		ANOVA and ANCOVA	100	Include
Kim et al. (2002)	Cross-sectional	Inclusion criteria: patients admitted to Korea University Medical Center emergency room consecutively after suicide attempt between January 1994 and July 2000 Exclusion criteria: panic disorder	Age, sex and BMI reported Korea University Medical Center emergency room	All subjects fasted overnight. Samples of blood were drawn from the antecubital vein in a sitting position using a tourniquet between 7 a.m. and 8 a.m. For the suicidal patients, blood was collected within 48 hours after the suicide attempt. Five millilitres of blood was collected into a vacuum tube with no additives. Total serum cholesterol levels were measured by enzymatic procedures using a Hitachi 717 analyser	DSM-III-R classification used	Age, gender and BMI identified as confounders	Confounders controlled in ANOVA analysis	The severity of suicide attempt was evaluated according to the degree of resulting medical injury on the 5-point Medical Lethality Rating Scale (A.T. Beck, unpublished)	ANOVA and *t*-test used	100	Include
Steinert et al. (1999)	Cross-sectional	Unclear, only ‘patients on general psychiatric admission unit’ stated	Age, age at admission, sex recorded General psychiatric admission unit in Germany	Blood samples taken within first 3 days after admission under sober conditions in the morning. TC determined in an external laboratory centre providing continuous quality controls	ICD-10 classification used	Potential confounding factors, such as BMI, smoking, medication, involuntary admission, not identified	No strategy for dealing with confounders	Aggression assessed after discharge using MOAS, SDAS, SOAS, and VS	Spearman’s rank for correlations used	62.5	Exclude
Kunugi et al. (1997)	Cross-sectional	Inclusion criteria: TC measured immediately after admission (within 24 hours), blood sampling carried out in the morning of the day following admission, total protein level and red blood cell count simultaneously measured Exclusion criteria: diagnosis of substance abuse or organic brain syndrome	Age, sex, psychical condition (injury, burn, intoxication or hypoxia) reported Emergency ward at Teikyo University Hospital, Tokyo, Japan	TC measured immediately after admission (within 24 hours), blood sampling carried out in the morning of the day following admission	DSM-II III-R classification used	States that ‘potential confounders were adjusted for’ but no specification of what these were	Confounders controlled in ANCOVA analysis	Cases consisted of suicide attempters that were discharged alive	ANCOVA and Student’s *t*-test used	87.5	Include

ICD: International Classification of Diseases; TC: total cholesterol; HDL: high-density lipoprotein; LDL: low-density lipoprotein; TG: triglyceride; ICD-10: International Classification of Diseases, 10th Revision; BMI: body mass index; TOP: Thematically Organised Psychosis study; DSM-IV: *Diagnostic and Statistical Manual of Mental Disorders* (4th ed.); IDS-C: Inventory of Depressive Symptomatology; HBL: hypobetalipoproteinaemia characterised by LDL lower than fifth percentile for age and sex; SOAS-R: Staff Observation Aggression Scale–Revised; DSM-IV-TR: *Diagnostic and Statistical Manual of Mental Disorders* (4th ed., text rev.); SIS-MAP: Scale for Impact of Suicidality – Management, Assessment and Planning of Care; VLDL: very low-density lipoprotein; MOAS: Modified Overt Aggression Scale; IRS: Impulsivity Rating Scale; BSI: Beck Scale for suicidal ideation; OPCRIT: Operational Criteria for Psychotic Illness checklist; ANOVA: analysis of variance; ANCOVA: analysis of covariance; DSM-III-R: *Diagnostic and Statistical Manual of Mental Disorders* (3rd ed., rev.); SDAS: Social Dysfunction and Aggression Scale; SOAS: Staff Observation Aggression Scale; VS: Violence Scale; OPD: Out-patient department; LPT:Lipid panel test.

Case–control studies were scored out of 10, based on the following criteria: were the groups comparable, other than the presence of disease in cases or the absence of disease in controls; were cases and controls matched appropriately; were the same criteria used for identification of cases and controls; was exposure measured in a standard, valid and reliable way; was exposure measured in the same way for cases and controls; whether confounding factors were identified; whether strategies to deal with confounding factors were stated; whether outcomes were assessed in a standard, valid and reliable way for cases and controls; was the exposure period of interest long enough to be meaningful; and whether appropriate statistical analyses were used.

Similarly, case series studies were scored out of 10. A point was given if: there were clear criteria for inclusion in the case series; the condition was measured in a standard, reliable way for all participants included; valid methods were used for identification of the condition for all participants included; the case series had consecutive inclusion of participants; the case series had complete inclusion of participants; there was clear reporting of the demographics of the participants in the study; there was clear reporting of clinical information of the participants; the outcomes or follow-up results of cases were clearly reported; there was clear reporting of the presenting site/clinic demographic information; and statistical analysis chosen was appropriate.

Finally, cross-sectional studies were scored out of 8, based on criteria assessing whether: the inclusion criteria were clearly defined; the study subjects and setting were described in detail; the exposure was measured in a valid and reliable way; objective, standard criteria was used for measurement of the condition; confounding factors were identified; strategies to deal with confounding factors were stated; the outcomes were measured in a valid and reliable way and appropriate statistical analysis was chosen. Studies excluded at this stage consistently lacked the identification of confounding factors and strategies to handle them appropriately. A list of articles with reasons for exclusion following quality appraisal is provided in Tables 1–3.

A data extraction table was created with the final selection of articles (Table 4), compiling data on: the first author’s name, year of publication, location of the study, study design, psychiatric conditions included in the study, the diagnostic classification used, sample size (and sex distribution), the number of schizophrenia, schizoaffective and psychotic patients included (and sex distribution), details of the case and control groups, form of cholesterol examined (TC, LDL, HDL, TG), findings classified by each aggression type (physical against self, physical against others, physical against objects, verbal) and any other findings (see Table 4).

**Table 4.** Details of the included studies and key findings.

Study	Location	Study design	Psychiatric conditions included in study	Diagnostic classification	Total sample size (gender distribution)	Number of schizophrenia/schizoaffective/psychotic patients and gender distribution (male:female)	Case control groups	Age of participants (years) Range and mean ± SD (if available)	Cholesterol measure/s examined in relation to aggression	Findings classified by aggression type				Other findings
										Physical against self	Physical against others	Physical against objects	Verbal	
Sankaranarayanan et al. (2020)	Database of patients with psychotic disorders from Australia	Cross-sectional	Schizophrenia, schizoaffective disorder	ICD-10	802 (540 males, 262 females)	591 schizophrenia 211 schizoaffective disorder	Cases – patients with schizophrenia or schizoaffective disorder Controls – values from the normal population	37.72 ± 10.92	TC HDL LDL TG Mean TG/HDL ratio	Low HDL more likely to report current suicidal ideation (OR = 0.375, 95% CI = [0.14, 0.99])				Confounders like psychological stress, impulsivity or serum cortisol were not included in the analysis. The HDL significance could be a type I error. The study was also conducted predominantly on community-dwelling patients with schizophrenia, not on acutely ill or inpatients with schizophrenia
Hjell et al. (2020)	Inpatient and outpatient clinics of major hospitals in Oslo, Norway	Cross-sectional	Schizophrenia spectrum disorder Bipolar spectrum disorder	DSM-IV	1001 (525 males, 476 females)	601	Cases – patients with schizophrenia spectrum disorder Controls – values from the normal population	>18 Median age for patients with no aggressive symptoms (NAS) 30. Median age for patients with medium level aggressive symptoms (MLAS) 29. Median age for patients with high level aggressive symptoms (HLAS) 25	TC LDL HDL		No relationship between TC, LDL or HDL and aggression, as measured by PANSS-EC (sum of items measuring excitement, hostility, tension, uncooperativeness and poor impulse control)			Highest levels of aggression scarcely represented among study participants. Median age of patients in sample was 29, below the recommended age of 40 for initiation of cardiovascular disorder risk evaluation and statin therapy initiation
Gohar et al. (2019)	Major hospitals in Oslo, Norway	Cross-sectional (with correlational analysis)	Schizophrenia Schizoaffective disorder Schizophreniform disorder Psychosis NOS	DSM-IV	270	270 (161 males, 109 females)	Cases: patients with mild/moderate suicidal behaviour and severe suicidal behaviour with/without suicide attempts Controls – patients without suicidal behaviour	18–65 years 30.7 ± 10.1	TC LDL TG	Negative but non-significant and small correlations between TC (*r* = −0.1) and LDL (*r* = −0.1), and suicidal behaviour when examined across the entire sample. No correlation between TG and suicidal behaviour. Data on group differences (those with mild/severe suicidal behaviour vs no suicidal behaviour) in TC and LDL not reported				Lower levels of leptin significantly increased the risk of being in the mild to moderate or severe suicidal behaviour groups (OR = 0.4, 95% CI = [0.2, 2.8]; OR = 0.5, 95% CI = [0.3, 0.8], respectively). Levels of TC and LDL significantly positively correlated with leptin (all *r* = 0.20; *p* < 0.01) across the entire sample, and not examined (i.e. not entered into the regression model) as predictors of membership to group with mild/severe suicidal behaviour.
Cariou et al. (2018)	Nantes University Hospital, France	Cross-sectional	• Schizophrenia, schizotypal and delusional disorders • Organic mental disorder • Mental disorders due to psychoactive substance use • Unspecified psychosis • Affective disorders • Neurotic disorders • Disorder of adult personality and behaviour Pervasive and specific developmental disorders	ICD-10	837 (of 839, 2 patients excluded due to secondary causes of HBL) (495 males, 342 females)	300 (male:female ratio not provided)	Cases: patients Controls: values from the normative population	>18 HBL: 35 ± 10 Non-HBL: 44 ± 14	LDL (calculated according to the Friedewald formula)	No association found between low LDL and suicide attempts or self-injuries across the whole sample	Not assessed	Participants with low LDL (⩽50 mg/dL) characterised by a greater number of schizophrenia patients (*p* = 0.044) and hetero-aggression (*p* = 0.015) (aggression directed at external objects)	Not assessed	
Eriksen et al. (2018)	Oslo University Hospital, Norway	Cross-sectional with longitudinal component	Psychosis Bipolar disorder Substance abuse Depression Anxiety Personality disorders	ICD-10	Inpatient sample 348 (156 males, 192 females in inpatient sample) Inpatients: follow-up sample 101 (47 males, 54 females)	Psychosis Group Inpatient 102, follow-up 29	Cases – patients admitted to an acute psychiatric ward Controls – values from the normal population	Inpatient: 18–83 Follow-up sample: 20–75	TC HDL		TC link with violence was non-significant for men and women for inpatients. HDL contributed to violence risk for men in the inpatient sample even when controlled for other variables (*p* = 0.032) but not women			
Capuzzi et al. (2018)	Psychiatric Inpatient Unit at Desio Hospital, ASSD Monza, Italy	Cross-sectional	Schizophrenia spectrum Type I or Type II bipolar Major depression Personality disorder	ICD-10	593 (328 males, 265 females)	195	Cases – patients with schizophrenia Controls – values from the normal population	>18 40.7 ± 12.1 years	TC LDL TG	No association between recent suicide attempts and TC (*p* = 0.841), LDL (*p* = 0.682) and TG (*p* = 0.515) in patients with schizophrenia				Number of suicide attempts was relatively small Most subjects were treated with pharmacological agents, including antipsychotic, mood stabilisers and antidepressants, which might influence lipid profile and its relationship with suicidal behaviours
Eriksen et al. (2017)	Oslo University Hospital, Norway	Cross-sectional with a longitudinal component	• Psychosis • Substance abuse • Bipolar disorder • Depression • Anxiety • Personality disorders	ICD-10	362 inpatients (158 males, 204 females); of these, 99 followed up at 3 months after discharge	104 (male:female ratio not provided)	NA	>18 18–95 Baseline inpatient sample: Mean = 41 Follow up outpatient sample, (outpatients) Mean 38	TC HDL	Not assessed	59 (16%) inpatients 26 (26%) post-discharge	Not assessed	Findings not stated	At inpatient assessment, a statistically significant association, OR [95% CI] = 0.52 [0.28, 1.0], *p* = 0.049) between low HDL and aggression (physical against others and verbal assessed together) At post-discharge assessments, no association across the entire sample (*p* = 0.186) but a significant association between low HDL and post-discharge violence, OR [95% CI] = 0.099 [0.010, 0.95], *p* = 0.045 when examined in men For psychosis (baseline inpatient assessment) and HDL, for inpatients, OR [95% CI] = 1.35 [1.28, 1.43], *p* < 0.001. No statistically significant association found between TC and aggression
Shrivastava et al. (2017)	A tertiary general psychiatric centre (hospital name not provided), in India	Cross-sectional, cohort study. Categorical observation	Non-affective schizophrenia	DSM-IV	60 (41 males, 19 females)	60 (41 males, 19 females)	NA	18–39	TC	Lower levels of TC (*p* = 0.047) in female patients with moderate suicidality (*n* = 9) compared to those with low (*n* = 6) or high suicidality (*n* = 4)	Not assessed	Not assessed	Not assessed	An association was not found for men between low TC and suicidality. No association for the whole sample between low TC and suicidality. Even for females, no association was found with suicidality, but this could be due to low numbers with high suicidality Correlation between TC and SIS-MAP scales (Scale for Impact of Suicidality. – Management, Assessment and Planning of Care) was non-significant (*r* = 0.203, *p* = 0.119). This study suggests that the relationship between TC and suicidality may not be necessarily linear. The sample size, however, was really small, even for moderate suicidality
Kavoor et al. (2017)	Tertiary level referral centre and post-graduate teaching hospital in western Uttar Pradesh, India	Cross-sectional. Categorical observation compared to age, sex and ethnicity matched healthy controls	Paranoid and undifferentiated schizophrenia	ICD-10	120 (92 males, 28 females)	60 (46 males, 14 females)	Cases: 60 adult schizophrenia patients 60 age- and sex-matched healthy controls	Cases: 32.40 ± 6.6 Controls: 32.42 ± 6.7	TC TG HDL LDL VLDL	A significant negative correlation (*r* = −0.303; *p* < 0.05) between TC and suicidal ideation, and LDL with suicidal ideation (*r* = −0.257, *p* < 0.05) assessed with the BSI in patients	A negative trend-level correlation (*r* = −0.183) between TC and physical aggression against others assessed through the MOAS score, though not statistically significant. Negative correlation between TC and impulsivity assessed using the IRS (*r* = 0.517, *p* < 0.01). Negative correlation between LDL and impulsivity (IRS) (*r* = 0.387, *p* < 0.01) but not aggression (MOAS) (*r* = 0.092) .There was also a negative correlation between TG and IRS-assessed impulsivity (*r* = 0.282, *p* < 0.05)	Findings not stated	Findings not stated	TC was significantly lower in the patient group compared to healthy controls (*p* < 0.001). HDL and LDL were also significantly lower in the patient group (*p* = 0.005 and *p* = 0.009). VLDL was also lower but not to statistical significance (*p* = 0.119) and so was TG (*p* = 0.068). Male schizophrenia patients additionally had lower TG levels than the healthy male control group (*p* = 0.025). TC showed negative correlation with impulsivity (*r* = 0.517, *p* < 0.01) and suicidality (*r* = 0.303, *p* < 0.05)
Chen et al. (2015)	National Taiwan University Hospital, Taiwan	Case series. Correlational observation	Schizophrenia	DSM-IV	107 (33 males, 74 females)	107 (33 males, 74 females) Patients classified into four trajectory classes (subgroups) based on their violence over the course of their hospitalisation Class 1: no violence (27 females, 13 males) Class 2: low-levelling off (patients with initial low violence score that decreased gradually) (25 females, 17 males) Class 3: high-falling sharply (patients with high initial violence scores that dropped rapidly and substantially before levelling off) (10 females, 1 male) Class 4: high-falling slowly (patients with highest violence scores that declined the most slowly) (12 females, 2 males)	NA	>16 33.4 (11.9)	TC TG LDL HDL	Findings not stated	Four subgroups (with different violence trajectories) did not differ (at entry) in TC, LDL or HDL There was a marginal difference in TG (0.06) with highest levels in lower levels in ‘no violence’ group and the lowest level in ‘high-falling sharply/high-falling slowly’ groups	Findings not stated	The major manifestation of violence in this study was verbal aggression towards others	Cases with an increased level of overall aggression exhibited a trend towards smaller proportion of high LDL level (*p* = 0.06). Female gender and low TC levels (along with early onset, higher scores of positive symptoms and lower scores of negative symptoms) were found to be predictors of violence with a predictive accuracy of 0.85 [95% CI] = [0.72, 0.97], *p* < 0.0001). Smaller sample sizes in the higher violence trajectory classes (3 and 4) might have prevented their association with lipid levels reaching statistical significance
Misiak et al. (2015)	Lower Silesian Centre of Mental Health, Wroclaw, Poland	Cross-sectional cohort study	Schizophrenia	DSM-IV	100 (53 males, 47 females)	100	Cases – 30 first-episode schizophrenia with lifetime experiences of suicidal ideation Controls – 70 first-episode schizophrenia patients with no lifetime experience of suicidal ideation	Range: 18–43 Males: 25.56 ± 4.89 s Females: 30.32 ± 7.91	TC (mg/dL) LDL (mg/dL) HDL (mg/dL) TG (mg/dL)	Association found between higher TC and suicidal ideation in females (*p* = 0.012), and between higher LDL and suicidal ideation in females (*p* = 0.020). This was based on group differences between cases and controls. No such differences found in males				This is offering a finding which is an opposite result to other studies mostly showing an association between low TC and suicidal risk, thus suggesting that the nature of lipid metabolism in patients experiencing suicidal ideation might be complex
Park et al. (2013)	Psychiatric ward at University Hospital in Seoul, Korea	Cross-sectional	Schizophrenia Bipolar affective disorder Major depressive disorder	ICD-10	516 (males 252, females 264)	246	Cases – patients with schizophrenia who died by suicide Controls – with schizophrenia who were non-suicidal	Schizophrenia patients with suicide: 29.1 ± 9.3 Controls (schizophrenia patients with non-suicide): 29.3 ± 8.9	TC TG HDL	No difference between patients who died by suicide and controls in schizophrenia, including TC, triglyceride and HDL cholesterol				No standardised procedure for arriving at diagnoses Information on other risk factors like symptomatic characteristics and poor adherence to medication not available
Turkoglu et al. (2009)	Elazig Mental Hospital, Turkey	Case control. Categorical observation between schizophrenia patients with and without a record of crime	Schizophrenia	DSM-IV	100 (all male)	100 (all male)	Cases – 50 male, criminal schizophrenia patients Controls – 50 male, non-criminal schizophrenia patients	19–59	TC TG Ghrelin	Not assessed	72% of crime included in the study were physical aggression against others, including homicide, assault and battery. Levels of serum TC were numerically lower in the schizophrenia patients who had committed a crime compared to those who had not but the difference was not statistically significant. (*p* > 0.05; exact value not reported). Serum TG levels and ghrelin were higher in the offender group, compared to the non-offender group (*p* < 0.001)	14% of crime included damage to public property	Not assessed	
Marcinko et al. (2008)	University Hospital Zagreb, Croatia	Cross-sectional	Schizoaffective disorder	ICD-10	60 (all males)	40 (20 with suicidal and 20 non-suicidal)	Cases – consecutively admitted patients with schizoaffective disorder with suicidal behaviour and consecutively admitted patients with schizoaffective disorder without suicidal behaviour Controls – age-matched healthy males with no history of psychiatric illness and somatic disorders	>18 Suicidal patients: 28.9 0 ± 8.76 Non-suicidal patients: 37.35 ± 12.21 Controls: 29.85 ± 7.57	TC TG HDL LDL	Suicidal patients had significantly lower levels of TC (*p* = 0.000) and LDL (*p* = 0.001) than non-suicidal patients. TG and HDL values were also lower but without a statistically significant difference				The authors claimed that this was the first study to show the relationship between reduced TC and suicidality in schizoaffective disorder
Ruljancic et al. (2007)	Sveti Ivan Psychiatric Hospital, Croatia	Case series. Categorical observation between psychiatric patients	• Schizophrenia • Schizoaffective disorder • Psychotic disorder • Dependence Syndrome • Depressive disorder • Stress reaction • Personality disorder • Non-violent suicidal attempt (drug poisoning) • Violent suicidal attempt (hanging, suffocation, sharp object)	ICD-10	677 (415 males, 262 females)	Schizophrenia: 136 (58 females, 78 males) Schizoaffective: 64 (49 females, 15 males) Psychotic disorders: 47 (19 males, 28 females)	NA	17–89	TC	Patients with a history of non-violent suicidal attempt (drug poisoning) had significantly lower serum TC than those with schizophrenia (*p* = 0.039) or schizoaffective disorder (*p* = 0.019)	Not assessed	Not assessed	Not assessed	There was a similar difference between non-violent suicidal attempters and depressive disorder, stress reaction and personality disorders. This study did not examine TC levels in relation to any history of violence within schizophrenia, schizoaffective/psychotic disorder groups
Marcinko et al. (2007)	Clinical Hospital Centre, Zagreb, Croatia	Cross-sectional with longitudinal follow-up over 12 months	Psychotic Disorder Not Otherwise Specified (first episode of psychosis)	DSM-IV-TR	81 (all males)	27	Cases – 27 consecutively admitted suicidal men in the first episode of psychosis and 27 consecutively admitted men in first episode of psychosis without suicidal behaviour Controls – healthy males with no history of psychiatric illness and suicidal behaviour	Suicidal patients: 29.70 ± 7.22 Non-suicidal patients: 29.56 ± 8.67 Healthy controls: 30.33 ± 3.13	TC	TC levels were significantly reduced in suicidal when compared to non-suicidal or healthy subjects, even after controlling for age and BMI (*p* = 0.028)				Platelet 5-HT concentration was significantly lower in suicidal than in non-suicidal patients or healthy controls (*p* < 0.001 and *p* = 0.002, Scheffe’s test, respectively)
Marcinko et al. (2005)	Clinical Hospital Centre Zagreb, Croatia	Case control. Categorical observation between schizophrenia patients with violent, non-violent and no suicidal attempts (control group)	Schizophrenia	ICD-10	46 (all males)	46 (all males)	Cases – 31 males suffering from schizophrenia, admitted after suicide attempt (15 non-violent attempters, 16 violent attempters) Controls – 15 schizophrenia non-suicidal male patients	Cases: 32.02 ± 8.21 Controls: 30.95 ± 9.03	TC Serum cortisol	Significantly lower TC in violent attempters compared to non-violent attempters (*p* < 0.01) and compared with no suicide controls (*p* < 0.01). No significant difference between the non-violent suicide attempters and no suicide controls	Not assessed	Not assessed	Not assessed	Serum cortisol concentration significantly higher (*p* < 0.01) in violent compared to non-violent suicide attempters, and no suicide attempters
Chakrabarti et al. (2004)	Central Institute of Psychiatry, Ranchi, India	Case control Categorical observation between psychotic patients with a history of violent crime and without a history of violent crime	• Schizophrenia (paranoid and undifferentiated) • Bipolar affective disorder • Recurrent depressive disorder	ICD-10	60 (all males)	46 – 23 in case group and 23 in control group, as the control group was matched to the case group by diagnosis (all males)	Cases – 30 male inpatients diagnosed with a psychotic illness with a history of violent crime Controls – 30 age and BMI matched psychotic inpatients with no history of violent crime	Cases: 33.17 ± 7.53 Controls: 32.43 ± 9.14	TC HDL LDL VLDL TG Apolipoprotein A1 Apolipoprotein B	Significant positive correlation between HDL and suicidality (*r* = 0.2113, *p* = 0.021)	Violent crime was assessed in this study and included homicide, rape, arson and grievous injury. Group with a history of violent crime showed significantly lower TC (*p* = 0.001) and lower LDL (*p* < 0.001) compared to group without a history of violence	Findings not stated	Not assessed	Though there was no significant association between higher HDL and TC in this study, higher HDL is known to be associated with lower total cholesterol Grandiosity (*p* = 0.012), elated mood (*p* = 0.039), motor hyperactivity (*p* = 0.031) and distractibility (*p* = 0.033) showed a negative correlation with APA1. Higher APA1 is associated with lower total cholesterol
Tripodianakis et al. (2002)	Evangelismos General Hospital, Greece	Case control. Categorical observation between patients and healthy controls (no psychiatric history), matched for age and sex	• Schizophrenia • Adjustment disorder • Depression • Personality disorder • All admitted to medical wards after a suicidal attempt	DSM-III-R	173 (66 males, 107 females)	16	Cases – 111 (35 males, 76 females) Controls – 62 healthy controls (no psychiatric history or history of suicide attempt) (31 males, 31 females)	Schizophrenia group: 30.4 ± 9.9 Controls: 31.6 ± 8.1	TC	Violent suicide attempters had a significantly lower TC level compared to controls (*p* = 0.041) and so did non-violent attempters (*p* = 0.0004) The difference between violent and non-violent suicide attempters was not significant (*p* = 0.94)	Not assessed	Not assessed	Not assessed	Significantly lower TC in the subgroup with schizophrenia (*p* < 0.02) (as well as adjustment disorder (*p* < 0.011) and personality disorder (*p* < 0.0001) compared to healthy controls
Repo-Tiihonen et al. (2002)	Niuvanniemi Hospital, Finland	Case control Categorical observation between patients who had been secluded involuntarily (for violent acts, for suicidal acts) and patients who had not been secluded	Schizophrenia, paranoid and other types Personality disorder Other	ICD-10	409 (all males)	226 (all males)	Cases – male secluded patients (207 total, of which 165 schizophrenia patients) Controls – male non-secluded patients (202 total, of which 61 schizophrenia patients)	15–71	TC	Low (below 5.3 mmol/L) TC level was deemed to be a marker (*p* = 0.011) for increased risk of violent and suicidal behaviour in these forensic patients	Findings not stated	Findings not stated	Findings not stated	Patients who had been secluded as a result of violent behaviour (*p* = 0.003) and suicidal acts (*p* = 0.026) had significantly lower TC levels than those who had not been secluded
Kim et al. (2002)	Korea University Medical Centre, Korea	Cross-sectional	Schizophrenia Bipolar disorder Personality disorder Major depressive disorder	DSM-III-R	693 (327 males, 366 females)	64 (40 males and 24 females)	Cases – suicide attempters with schizophrenia (*n* = 231) Controls – non-suicidal psychiatric inpatients (*n* = 231)and normal healthy controls (*n* = 231)	Cases: age not specified but mean age for the whole patient group 38.1, with SD 17.0 Controls: age not specified but mean age for psychiatric controls: 38.2 ± 15.4; normal control: 38.2 ± SD 16.3	TC	Risk to self – no significant difference in serum TC levels found in suicide attempters with schizophrenia compared to non-suicidal attempts as well as normal controls				Total serum TC levels among suicide attempters was significantly lower (*p* < 0.001) in patients with major depressive disorder and personality disorder, but not also in bipolar disorder
Huang et al. (2000)	Chang Gung Memorial Hospital, Kaohsiung, Taiwan	Case series. Categorical analysis – between paranoid and non-paranoid schizophrenic patients	Schizophrenia Bipolar I disorder (mania) Major depression Organic mental disorder Drug dependence Alcohol dependence Delusion disorder Others	DSM-III-R	213 (gender distribution not specified)	106 (47 males, 59 females)	Cases – psychiatric patients with any kind of diagnosis. Controls – normal population	30.6 ± 10.8	TC	No significant differences (*p* > 0.80) in the serum TC levels between patients who had (*n* = 14) or had not made a suicidal attempt (*n* = 92)	No significant differences (*p* = 0.8373 > 0.05) in the serum TC levels between patients with physical violence (*n* = 22) and without (*n* = 84)	Not assessed	Not assessed	No significant difference (*p* = 0.96) in mean serum TC levels between men and women No significant differences (*p* > 0.24) in mean serum TC between paranoid and no-paranoid sub-types Serum TC levels were lower (*p* < 0.001) in psychiatric inpatient group compared to controls
Kunugi et al. (1997)	Tokyo University Hospital, Tokyo	Cross-sectional	Schizophrenia spectrum disorders Mood disorders Personality or neurotic disorders	DSM-III-R	173 (99 males, 74 females)	66	Cases – patients with schizophrenia admitted with suicide attempt Controls – patients with schizophrenia without suicide attempt	37 suicide attempters: age not specified but mean age for whole sample: 40 ± 16.7 29 non-suicide attempters: age not specified but mean age for whole sample 39.6, with SD 15.5	TC	No significant difference in TC levels between suicidal and non-suicidal groups in schizophrenia spectrum disorders				Significantly lower TC in suicide attempters than controls with mood disorders and with personality or neurotic disorders

ICD-10: International Classification of Diseases, 10th Revision; TC: total cholesterol; TG: triglyceride; HDL: high-density lipoprotein; OR: odds ratio; CI: confidence interval; DSM-IV: *Diagnostic and Statistical Manual of Mental Disorders* (4th ed.); LDL: low-density lipoprotein; HBL: Hypobetalipoproteinaemia characterised by LDL lower than fifth percentile for age and sex; VLDL: very low-density lipoprotein; BSI: Beck Scale for suicidal ideation; MOAS: Modified Overt Aggression Scale; IRS: Impulsivity Rating Scale; DSM-IV-TR: *Diagnostic and Statistical Manual of Mental Disorders* (4th ed., text rev.); BMI: body mass index; DSM-III-R: *Diagnostic and Statistical Manual of Mental Disorders* (3rd ed., rev.); APA 1: Apolipoprotein A1; PANSS-EC: Positive and Negative Syndrome Scale–Excited Component.

## Results

**Figure 1.**
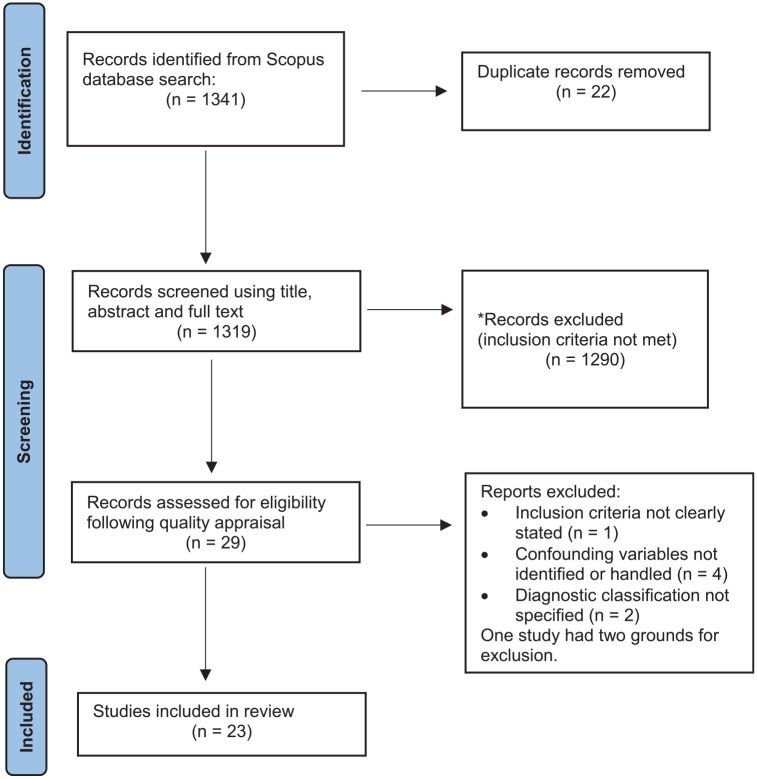
Flow chart of study selection.

Our literature search produced 29 articles for quality appraisal, of which 23 were deemed eligible for inclusion (Figure 1).

### Studies on suicide

In total, 20 studies (see Table 4) examined attempted suicide and suicidal behaviour, and some of these studies considered violent suicide attempts in inpatients with schizophrenia.

Eight of these 20 studies (Kavoor et al., 2017; Marcinko et al., 2005, 2007, 2008; Repo-Tiihonen et al., 2002; Ruljancic et al., 2007; Sankaranarayanan et al., 2020; Tripodianakis et al., 2002) concluded that relatively lower levels of cholesterol were associated with suicidality in patients with schizophrenia. Additionally, one of these studies (Repo-Tiihonen et al., 2002) found relatively lower TC (below 5.3 mmol/L) to be a marker of violent and suicidal behaviour. Apart from TC, low levels of HDL were associated with suicidality in three studies (Kavoor et al., 2017; Marcinko et al., 2008; Sankaranarayanan et al., 2020), of which one study was on males with schizoaffective disorder (Marcinko et al., 2008). Two of these studies also showed a link of suicidality with low LDL (Kavoor et al., 2017; Marcinko et al., 2008). Of these eight studies, one small sample study (Marcinko et al., 2005) showed that patients with a violent suicidal attempt had significantly lower TC levels than patients with non-violent attempts and non-suicidal controls but another study (Tripodianakis et al., 2002) with a far larger sample size found no such difference. Yet another study (Ruljancic et al., 2007) found a link between relatively low cholesterol and non-violent suicidal attempts (across several disorders) when compared to those without a suicidal attempt but did not report on such a link specifically for schizophrenia, schizoaffective disorder or psychotic disorder.

A total of seven studies (Capuzzi et al., 2018; Cariou et al., 2018; Gohar et al., 2019; Huang and Wu, 2000; Kunugi et al., 1997; Park et al., 2013; Shrivastava et al., 2017) did not find any link between lipid profile and suicidality in schizophrenia. In addition, one study (Chen et al., 2015) that examined the four types of aggression did not report findings on suicidality. One study (Misiak et al., 2015) found the opposite link, where relatively higher TC was associated with suicidal ideation in females with first-episode schizophrenia, thus demonstrating the complexity of the link. Finally, one study (Kim et al., 2002) found a link between relatively low TC and suicidality for major depressive disorder and personality disorder, but not for schizophrenia.

With regard to TG, seven studies (Capuzzi et al., 2018; Gohar et al., 2019; Kavoor et al., 2017; Marcinko et al., 2007; Misiak et al., 2015; Park et al., 2013; Sankaranarayanan et al., 2020) explored TG and suicidality, and none found an association.

All these studies were spread over a time period of more than two decades between 1997 and 2020. Sample sizes varied widely, from 31 to 802, though the smaller studies targeted patients admitted to hospital following a suicidal attempt. It is interesting to note, however, that the study (Sankaranarayanan et al., 2020) with the largest sample size (*n* = 802) did find a link between lipid profile and suicidality.

### Studies on aggression against others

A total of nine studies (Cariou et al., 2018; Chakrabarti et al., 2004; Chen et al., 2015; Eriksen et al., 2017, 2018; Hjell et al., 2020; Huang and Wu, 2000; Repo-Tiihonen et al., 2002; Turkoglu et al., 2009) explored the link between lipid profile and violence against others, of which six studies found a link. However, there were some variations in the findings. One group of researchers (Eriksen et al., 2018) did not find a link with TC but with HDL, and only for men and not for women. The same authors, while studying violence in the first 3 months after discharge, also found HDL to be inversely associated with violence in men only (Eriksen et al., 2017). One study (Repo-Tiihonen et al., 2002) tried to identify a cut-off point for low TC levels to be a marker for violence, measured by frequency of seclusion, finding the cut-off level to be 5.3 mmol/L. Another researcher group (Kavoor et al., 2017) found a correlation with TC and LDL but not with HDL for measures of psychopathology, impulsivity and aggression (and suicidality).

One study (Cariou et al., 2018) retrospectively evaluated hospitalised patients over the course of a year and found a link between low plasma concentration of LDL and hetero-aggression. The group with low LDL also had a higher frequency of schizophrenia, thus reinforcing the link between low LDL levels, psychiatric disorders and aggression (Cariou et al., 2018).

Serum TG was explored in five studies (Cariou et al., 2018; Chakrabarti et al., 2009; Chen et al., 2015; Kavoor et al., 2017; Turkoglu et al., 2009) involving violence to others, with one finding a clear link between low TG and violence (Cariou et al., 2018), one between high TG and violence (Turkoglu et al., 2009), with the other three studies finding no significant link, but a trend towards a link between low TG and violence (Chakrabarti et al., 2009; Chen et al., 2015; Kavoor et al., 2017).

Two studies (Hjell et al., 2020; Huang and Wu, 2000) did not find a link between measures of serum cholesterol and violence in schizophrenia patients. Of these, the largest and most recent study (Hjell et al., 2020) had a large sample size (*n* = 1001) and examined all patients with schizophrenia recruited from inpatient and outpatient clinics of major hospitals in Oslo, Norway, between the years 2002 and 2017. It had 601 patients with schizophrenia spectrum disorders, and the measure of aggression was the PANSS Excited Component (PANSS-EC; Kay et al., 1987). This scale has items on excitement, hostility, tension, uncooperativeness and poor impulse control. Impulsivity was measured with the Barratt Impulsiveness Scale (BIS-11; Patton et al., 1995). There were no significant associations between TC, LDL, HDL and aggression or impulsivity in patients with schizophrenia spectrum disorders. The other study (Huang and Wu, 2000) examined paranoid and non-paranoid schizophrenia patients and did not find significant differences in serum cholesterol levels between patients with and without physical violence and patients who had or had not made a suicidal attempt. This study was on an exclusively inpatient sample.

The studies were again spread over a time period of almost two decades, between 2002 and 2020. Sample sizes varied widely, the largest study with a sample size of 1001 (Hjell et al., 2020), medium level studies with sample sizes of 409 and 348 (Eriksen et al., 2018; Repo-Tiihonen et al., 2002) and the smallest with a sample size of 60 patients (Chakrabarti et al., 2009).

### Studies assessing sex differences

For the studies on suicidality, there were only three studies and these were exclusively single sex, for males (Marcinko et al., 2005, 2007, 2008). One found a link between low TC, low LDL and low HDL (but not low TG) and suicidality in schizoaffective disorder (Marcinko et al., 2008), one found a link between low TC and suicidality in first-episode psychosis (Marcinko et al., 2007) and the third study found low TC in patients with violent suicidal attempts as opposed to non-violent suicidal attempts and controls (Marcinko et al., 2005).

For the studies exploring link between cholesterol levels and violence, there were three studies exclusively on males (Chakrabarti et al., 2004; Repo-Tiihonen et al., 2002; Turkoglu et al., 2009), all involving forensic populations. These studies all found a significant association between relatively low TC and violence, except one (Turkoglu et al., 2009), where the association did not reach statistical significance. With regard to the studies which found a positive link between low TC and suicidality, one study (Misiak et al., 2015) which had both males and females in it found a link between relatively higher TC and LDL levels with suicidal ideation in female, but not in male first-episode schizophrenia patients. One problem with this study, however, was the relatively low overall prevalence of suicidal ideation. The authors themselves advise caution in the interpretation of the study results.

With regard to the studies examining the link between lipid profile and violence, one study (Eriksen et al., 2018) found low HDL to be contributing significantly to the model for violence risk assessment in males, but not females. In another report from the same group (Eriksen et al., 2017), low HDL level was significantly correlated with violence for males but not females in the first 3 months following discharge. However, another study (Chen et al., 2015), where sex distribution was 33 male and 74 female schizophrenia patients, found female sex and low dichotomised TC levels as two of five variables (others being early onset, higher scores of positive symptoms and lower scores of negative symptoms) which could be used as predictors of violence, with a predictive accuracy of 0.85 (95% CI = [0.72, 0.97]).

The studies which exclusively examined male populations were all in forensic patients and all suggested a link between lower TC and violence (Chakrabarti et al., 2004; Repo-Tiihonen et al., 2002; Turkoglu et al., 2009). However, there have been no recent studies on forensic populations, and none involving female forensic populations.

A study of 41 male and 19 female schizophrenia patients (Shrivastava et al., 2017) observed that females with moderate but not low or high suicidality had lower cholesterol levels to statistical significance, but such a link was not found for males. However, sample sizes were really low (*n* = 4–9 per subgroup in female participants) in this study.

Overall, the link between lipid profile and violence seems to be slightly stronger for males than females, but that might well be because males have been studied more often than females. There is also some inconclusive evidence to suggest that the relationship might be different for females and males, both for violence and suicidality.

## Discussion

There were three main findings in relation to the primary objectives of this systematic review.

First, there were many more studies which reported on a link between low cholesterol and violence to others than those which did not find a link. All researchers investigating forensic samples found such a relationship. However, the relationship was not only with low TC but also with other parameters of the lipid profile, such as low LDL and low HDL. It is thus important not just to collect data on TC, but LDL and HDL as well. The evidence was weaker for the link between TG and violence, with only two studies finding a link, but one with high TG, the other with low TG. It is to be noted that the study with the largest sample size failed to find a link (Hjell et al., 2020). However, one of the issues in this study was the relatively low rate of baseline violence in the study population, thus offering some indication that the link is likely to be more valid in groups where there is a greater prevalence of violence (i.e. forensic populations).

The biological mechanism behind cholesterol–violence association has been hypothesised to involve serotonin. Animal studies have found an increase in violent behaviour in monkeys assigned to a low-cholesterol diet (Kaplan et al., 1991, 1994). Kaplan et al. (1991) suggested that low cholesterol may reduce cell viscosity and reduce serotonergic receptor activity. This may lead to a depressive state (Marcinko et al., 2005), increased impulsivity (Carver and Miller, 2006; Paaver et al., 2007) and aggressive behaviour (Chakrabarti et al., 2004). This finding was observed in Kaplan et al. (1994), where monkeys with experimentally lowered cholesterol had higher concentrations of serotonin metabolites than monkeys given high-cholesterol diets. However, the exact biological mechanism underlying cholesterol–violence (towards self/others) association in humans is still unclear.

Second, there was mixed evidence for a link between low cholesterol and suicidality in schizophrenia, with almost equal number of studies in favour and against such a link. The majority of studies used suicide as the method of assessing aggression. Eight studies found a statistically significant association between low cholesterol and suicide attempts, two of which only found this association in females (Misiak et al., 2015; Shrivastava et al., 2017). Seven studies failed to find a link. However, the study with the largest sample size (Sankaranarayan et al., 2020) did find a link, thus suggesting that methodological flaws, particularly low power, might have contributed to the variation in findings.

The evidence for a specific relationship between lipid profile and violent suicidal attempts was even more mixed. Ruljancic et al. (2007) found a significant association between low cholesterol and non-violent suicide attempts, in contrast to two other studies (Marcinko et al., 2005; Tripodianakis et al., 2002), reporting an association between low cholesterol and violent suicide attempts. While the two studies showing a positive association had smaller sample sizes, such findings are supported by other literature (Garland et al., 2000; Vevera et al., 2003). A meta-analysis found 50% more violent deaths in males with relatively low cholesterol than in participants with higher levels of cholesterol (Jacobs et al., 1992). This meta-analysis also found a higher prevalence of depressive symptoms in males with low serum cholesterol.

An association between low cholesterol and depression has frequently been observed (Grussu et al., 2007; Ploeckinger et al., 1996; Troisi et al., 2002). It is plausible that the causal pathway from low cholesterol to suicidality in schizophrenia patients involves depression. Kunugi et al. (1997) did not find a significant difference in cholesterol level between suicidal and non-suicidal inpatients with schizophrenia spectrum disorders. However, significantly lower cholesterol levels were found in suicide attempters with mood disorders compared with controls. Similarly, Modai et al. (1994) found a significant association between lower cholesterol and suicidality in patients with depression, but not in schizophrenia patients. Around 40% of people with schizophrenia have depression, with depression being the most significant factor in completed suicide (Upthegrove et al., 2016). People with schizophrenia and comorbid depression also have a significantly increased risk of violence and suicidality (Conley et al., 2007) and poorer clinical outcomes (Gardsjord et al., 2016; Upthegrove et al., 2009). The studies included in this systematic review did not control for depressive symptoms in schizophrenia patients. Although findings of low cholesterol and depression are inconsistent, with a number of researchers not finding a significant association (Cepeda et al., 2020; Van Dam et al., 1999), it is plausible that suicidality seen in schizophrenia patients with low cholesterol may be a consequence of depressive symptoms.

Third, most of the studies had similar findings for males and females. However, low HDL was seen in two studies to be a better predictor of violence than low TC for males (Eriksen et al., 2017, 2018). Another study found female sex and low TC levels could be used, in part, as predictors of violence in patients with schizophrenia (Chen et al., 2015). One study found higher TC and LDL levels to be associated with suicidality in females but not males with first-episode schizophrenia, thus suggesting that the link between lipids and violence is somewhat complex (Misiak et al., 2015). Future studies need to be designed to explore the nature of the link separately for females and males. All studies (Chakrabarti et al., 2004; Marcinko et al., 2005, 2007, 2008; Repo-Tiihonen et al., 2002) investigating only male schizophrenia patients found a significant association between low cholesterol and suicidality, except one group (Turkoglu et al., 2009) who did not report a significant association. On the contrary, three study authors observed an association only in female participants: two for suicidality (Misiak et al., 2015; Shrivastava et al., 2017) and one for violence towards others in patients with schizophrenia (Chen et al., 2015). However, one study (Eriksen et al., 2017) did not find this specific correlation in female participants.

Thus, it can be concluded that cholesterol levels could be used, in part, as predictors of violence in patients with schizophrenia. The evidence for a clear link with sex remains inconclusive. This is an important area for further work given that rates of violence in psychiatric patients following discharge tend to be higher in men than in women (Robbins et al., 2003). In addition, the neurobiology of violence, factors like body mass index (BMI), incidence of metabolic syndrome following antipsychotic medication and the effects of cholesterol are different between men and women, suggesting that discrete effects may occur in each sex (Golomb, 1998).

### Limitations

There were several limitations regarding the studies reviewed. First, in many of the studies reviewed, the main focus was on suicide and physical aggression to others. As a result, most patients observed were clinically ill enough to warrant inpatient treatment. Few studies evaluated aggression as physical against property and verbal, potentially deemed less severe and more likely to be seen in community or outpatient-based studies. A representative view of schizophrenia patients of all severities, including inpatients and outpatients, was therefore not specifically assessed in this review. Second, confounding factors, including sex, medication and age, may have affected the findings of this review. According to the Gender Paradox in Suicide, men are more likely to die by suicide than women, while women are more likely to engage in suicidal behaviour and deliberate self-harm (Canetto and Sakinofsky, 1998). However, self-harm was not frequently assessed in these studies. There is some indication of a link between low TC, HDL, LDL and impulsivity, which could translate into risk of self-harm, but this link needs to be explored through studies in populations with higher self-harming behaviours, like inpatient female wards. Medication, such as certain antipsychotics like risperidone, stimulate serotonergic output in cortical and subcortical areas more than other antipsychotics (Amato, 2015), and this may contribute to changes in aggressive behaviour independent of cholesterol level. In addition, with age comes a natural variance in cholesterol level, with a tendency of increased serum cholesterol with increasing age (Morgan et al., 2016). Aggressive behaviour may be independent of low cholesterol in younger participants as they, typically, would have a lower cholesterol level when compared to older patients. The effects of age as well as medication were not accounted for in a number of studies. Finally, the lack of statistical analysis using a 95% confidence interval in a number of studies (see Table 4) limited the review by making it difficult to assess precision and risk in data obtained.

In addition, there are limitations to the review itself. First, the review may have been limited by only allowing diagnosis of schizophrenia by a variant of DSM or ICD classifications. This excluded foreign classifications such as T-SCL-90-R. Second, selection bias arose when only considering studies reported in English. The inclusion and exclusion criteria stated that participants must be adults only, unless unavoidable. As a result, three included studies (Chen et al., 2015; Repo-Tiihonen et al., 2002; Ruljancic et al., 2007) had some participants below the age of 18, leading to wide variations in the age range of the study participants.

## Conclusion

This systematic review presents encouraging evidence of low cholesterol showing promise as a biomarker of risk in patients with schizophrenia. The majority of studies (around 70%) did find an association between low cholesterol and violence towards others, as well as about half of the studies with suicidal behaviour. However, studies specifically focusing on males and females with schizophrenia were lacking, along with relatively small sample sizes for some studies. Future studies need to be carried out focused exclusively on participants with a diagnosis of schizophrenia, and with histories of violent, self-harming and suicidal behaviours. These studies need to be longitudinal, exploring measures of self-harm, suicidality and aggression in male and female patients, both inpatients and outpatients, while taking age, medication history and any comorbidities into account, to explore patterns of link with lipid profiles including TC, LDL and HDL. Preferably, these studies should also include patients with other diagnoses as comparison groups. Such research would be essential to explore how a relatively commonly assessed measure like serum cholesterol along with high- and low-density lipoprotein could be incorporated into calculations of risk for any patient with schizophrenia, whether it is risk to others or to self.
